# Hydroxycinnamic Acid Antioxidants: An Electrochemical Overview

**DOI:** 10.1155/2013/251754

**Published:** 2013-07-16

**Authors:** José Teixeira, Alexandra Gaspar, E. Manuela Garrido, Jorge Garrido, Fernanda Borges

**Affiliations:** ^1^CIQ/Departamento de Química e Bioquímica, Faculdade de Ciências, Universidade do Porto, 4169-007 Porto, Portugal; ^2^Departamento de Engenharia Química, Instituto Superior de Engenharia do Porto (ISEP), Instituto Politécnico do Porto, 4200-072 Porto, Portugal

## Abstract

Hydroxycinnamic acids (such as ferulic, caffeic, sinapic, and *p*-coumaric acids) are a group of compounds highly abundant in food that may account for about one-third of the phenolic compounds in our diet. Hydroxycinnamic acids have gained an increasing interest in health because they are known to be potent antioxidants. These compounds have been described as chain-breaking antioxidants acting through radical scavenging activity, that is related to their hydrogen or electron donating capacity and to the ability to delocalize/stabilize the resulting phenoxyl radical within their structure. The free radical scavenger ability of antioxidants can be predicted from standard one-electron potentials. Thus, voltammetric methods have often been applied to characterize a diversity of natural and synthetic antioxidants essentially to get an insight into their mechanism and also as an important tool for the rational design of new and potent antioxidants. The structure-property-activity relationships (SPARs) correlations already established for this type of compounds suggest that redox potentials could be considered a good measure of antioxidant activity and an accurate guideline on the drug discovery and development process. Due to its magnitude in the antioxidant field, the electrochemistry of hydroxycinnamic acid-based antioxidants is reviewed highlighting the structure-property-activity relationships (SPARs) obtained so far.

## 1. Introduction

In the last decade, dietary polyphenols, which are the most abundant antioxidants present in a human diet, have received increasing interest from researchers, food manufacturers, and also consumers as one of the most promising groups of dietary preventive agents. They constitute a heterogeneous family of chemical compounds comprising phenolic acids, flavonoids, tannins, stilbenes, coumarins and lignans, among others.

Phenolic acids are usually divided into two main groups: benzoic acids, containing seven carbon atoms (C6-C1), and cinnamic acids, comprising nine carbon atoms (C6-C3). These compounds exist predominantly as hydroxybenzoic and hydroxycinnamic acids and may occur either in their free or conjugated forms.

Cinnamic acids have been categorized as structural and functional constituents of plant cell walls and also as bioactive ingredients of the diet [1, 2]. Recent data support their beneficial application as preventive and/or therapeutic agents in several oxidative stress related diseases, such as atherosclerosis, inflammatory injury, and cancer [3, 4]. Data found in the literature shows that hydroxycinnamic acids antioxidant efficacy is strongly dependent on their structural features and intrinsically related to the presence of hydroxyl function(s) in the aromatic structure [4, 5].

## 2. Hydroxycinnamic Acids: Classification and Occurrence

Hydroxycinnamic acids (HCAs) possess a simple chemical backbone consisting of a phenylpropanoid C6-C3 structure and are the major subgroup of phenolic acids with ubiquitous distribution in the plant kingdom. They are abundantly found in tea leaves, coffee, red wine, various fruits (especially red ones), vegetables and whole grains.

Hydroxycinnamic acids, such as *p*-coumaric, caffeic, ferulic, and sinapic acids (Table 1), are known to play an important role in nature. In fact, their wide distribution and high concentration provide them with a key role in the biosynthesis of more complicated phenolic systems. HCAs are secondary metabolites that are found also in several conjugated forms, including amides (conjugated with mono- or polyamines, amino acids, or peptides), esters, mainly esters of hydroxy acids, such as tartaric acid and sugar derivatives, and glycosides. Whereas cinnamate esters occur widely in higher plants amides of cinnamic acids seem to be rare.

The variety of cinnamic derivatives in plants is dependent on the species. In general, plants of the Solanaceae family provide both chlorogenic acid (5-caffeoylquinic acid) and free hydroxycinnamic acids, such as caffeic acid. These compounds are the most predominant in many fruits constituting up to 75% of phenolic acids. Actually, derivatives of cinnamic acid exist in all fruit parts, but concentrations are higher in the outer parts of ripe fruit.

## 3. Hydroxycinnamic Acids: An Antioxidant Outlook

Antioxidants, used to prevent or inhibit the natural phenomena of oxidation, have a broad application in diverse fields as they have a huge importance either as industrial additives or health agents. In this context, HCAs have been ascribed to act as powerful antioxidant compounds possessing diverse physiological roles in biological systems. Research data have revealed that HCAs can be used for preventive and/or therapeutic purposes in several diseases related to oxidative stress (e.g., atherosclerosis, inflammatory injury, cancer, and cardiovascular diseases).

The antioxidant foremost mechanism of action is assumed to be through its radical-scavenging activity that is linked to their hydrogen- or electron-donating ability and to the stability of the resulting phenoxyl radicals. However, other mechanisms of action have been suggested such as chelation of transition metals, like copper or iron, which are well-known catalysts of oxidative stress, inhibition of ROS/RNS-generating enzymes, and modulation of gene expression (ARE/Nrf-2 pathway) [12–14].

Generally, cinnamic acids work well in aqueous media being their hydrophilic nature a restriction for lipophilic systems protection (e.g. food or cosmetic matrices or biological membranes) due mainly to the difficulty of their incorporation into fat and oil mediums. The hydrophobicity of HCAs can be enhanced by chemical modification, namely, by esterification of the carboxyl group of phenolic acid with, for instance, a fatty alcohol. Using this type of approach, one must take in mind that the original functional properties must be retained. Therefore, amplified sort of applications of these modified natural antioxidants in lipophilic medium can be explored.

## 4. Electrochemistry of Hydroxycinnamic Acids and Derivatives

### 4.1. General Concepts

Electrochemical techniques are versatile and powerful analytical tools, which are able to provide high sensitivity and low detection limits, associated with the use of inexpensive instrumentation. In addition to their application in fundamental studies of oxidation and reduction processes to unravel reaction mechanisms these techniques are also used in studying the kinetics and thermodynamics of electron and ion transfer processes [15, 16].

The common characteristic of all voltammetric techniques is that they involve the application of a potential (*E*) to an electrode which causes the species to be determined to react and a current (*I*) to pass. In many cases, the applied potential is varied or the current is monitored over a period of time (*t*). Thus, all voltammetric techniques can be described as some function of *E*, *I*, and *t*. They are considered active techniques (as opposed to passive techniques such as potentiometry) because the applied potential forces a change in the concentration of an electroactive species at the electrode surface by electrochemically reducing or oxidizing it [17]. 

Cyclic voltammetry (CV) has become an important and widely used electroanalytical technique in many areas of chemistry and biochemistry. It is rarely used for quantitative determinations, but it is widely used for the study of redox processes, namely, for obtaining information on the nature of intermediates and stability of reaction products. 

Advanced voltammetric techniques have been developed to increase the sensitivity and selectivity of the voltammetric signal. In most cases, this requires minimizing the current from nonfaradaic processes such as that due to the charging and discharging of the double layer [16, 18]. These techniques rely on combinations of sweeps and steps and have names such as normal pulse voltammetry, differential pulse voltammetry, and square wave voltammetry [16, 19].

Voltammograms show peaks and waves which represent the different redox processes occurring in the potential window selected. The positions of the peaks and waves along the potential axis reflect the species involved (via their redox potential) but also whether the redox reactions are kinetically favorable or unfavorable. The shape and height of the peaks and waves also indicate the nature of the redox processes (e.g., whether a given redox process involves an adsorption/desorption or whether both reactants and products are soluble). A voltammogram is therefore a kind of finger print of the redox reactions [20]. 

### 4.2. Electrochemistry and Antioxidant Assays

The term “antioxidant” is increasingly popular nowadays as it is associated with a number of health benefits. In fact, antioxidants are molecules that can be associated with the protection of macromolecules from oxidation [21]. In biological systems, the definition has been accepted to be “any substance that, when present at low concentrations compared to those of an oxidisable substrate, significantly delays or prevents oxidation of that substrate” [22, 23].

All termed dietary and biological antioxidants are believed to be effective compounds, and in general all of them exhibit a moderate-marked native electroactivity. Therefore, electrochemical techniques can play a relevant role in the evaluation of antioxidants' capacity.

**Scheme 1:**
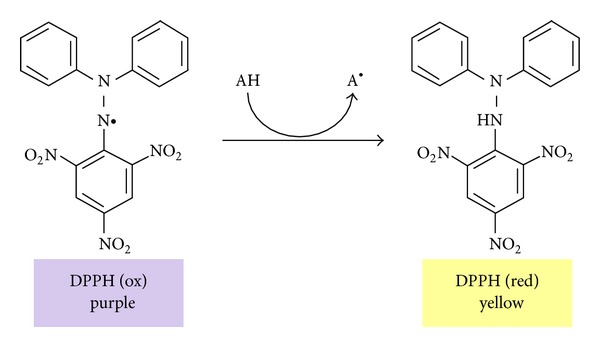
Principle of DPPH radical scavenging capacity assay.

Electrochemistry is the conceptual base of several antioxidant capacity assays. In general, the electron transfer (ET) based assays evaluate the capacity of an antioxidant to reduce an oxidant, which usually change color when reduced [24]. ET-based assays encompass one of the most popular antioxidant assays, the DPPH radical scavenging capacity assay (Scheme 1). 

Electrochemical approaches have been often used to evaluate the overall reducing power of antioxidant compounds present in food and biological samples. They can be considered a direct test for the evaluation of total antioxidant capacity of a diversity of compounds as they are based only on their chemical-physical properties simplifying notably the overall analytical process [25–27]. Actually, being the oxidation potential conceptually correlated with the antioxidant capacity, one can conclude that low oxidation potentials, found either in food or biological samples, enlighten a high antioxidant capacity. Additionally, the amperometric current and/or charge measured under fixed oxidation conditions can also provide an idea about the extension of their antioxidant capacity as well as the estimation of their total content [27]. 

### 4.3. Electrochemical Behaviour of Hydroxycinnamic Acids and Derivatives

Considering that hydroxycinnamic acids are antioxidants compounds by excellence, electrochemical techniques can be powerful tools for the study of reaction mechanisms involving electron transfer and afford complementary information. The main structural feature responsible for the antioxidant and free radical-scavenging activity of hydroxycinnamic acid derivatives is the number and location of hydroxyl groups present in the molecule. Phenols are able to donate the hydrogen atom of the phenolic group to the free radicals, stopping the chain propagation step during the oxidation process.

Differential pulse (DP) and cyclic voltammetry were used to investigate the oxidation of *p*-coumaric, caffeic and ferulic acids (Table 1). 

The first cyclic voltammetric scan of *p*-coumaric acid showed the occurrence of one irreversible peak which was associated with the oxidation of the hydroxyl group on the aromatic ring of the molecule [6, 28–31]. After successive oxidative scans, a *p*-coumaric acid oxidation product is deposited on the electrode surface forming a polymeric film [30]. A DP voltammetric study of *p*-coumaric acid was also performed over a wide pH range from 3.6 to 12.7. It has been concluded that the oxidation of *p*-coumaric acid below pH = pK_*a*_ occurs with the transfer of one electron and one proton [30]. The voltammetric behavior of *p*-coumaric acid is in agreement with the general mechanism proposed for oxidation of phenolic groups [32]. The general stage of oxidation of phenols leads to the formation of a phenolate ion that can be further oxidized to a phenoxyl radical. These products can undergo further chemical reactions such as coupling (polymers), proton loss, or nucleophilic attack [6, 28–31].

**Figure 1:**
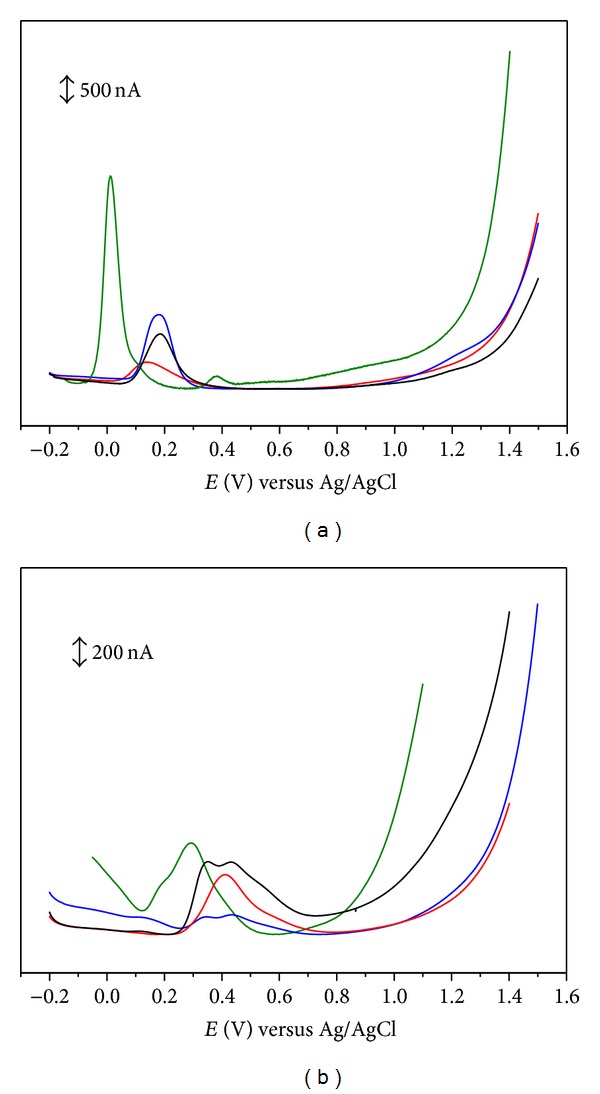
Differential pulse voltammograms for 0.1 mM solutions of (a) (black line) caffeic acid, (blue line) 5-bromocaffeic acid, (green line) 3,4,5-trihydroxycinnamic acid, and (red line) hydrocaffeic acid and (b) (black line) ferulic acid, (blue line) 5-bromoferulic acid, (green line) sinapic acid, and (red line) hydroferulic acid, in physiological pH 7.3 supporting electrolyte. Scan rate: 5 mV s^−1^.

**Figure 2:**
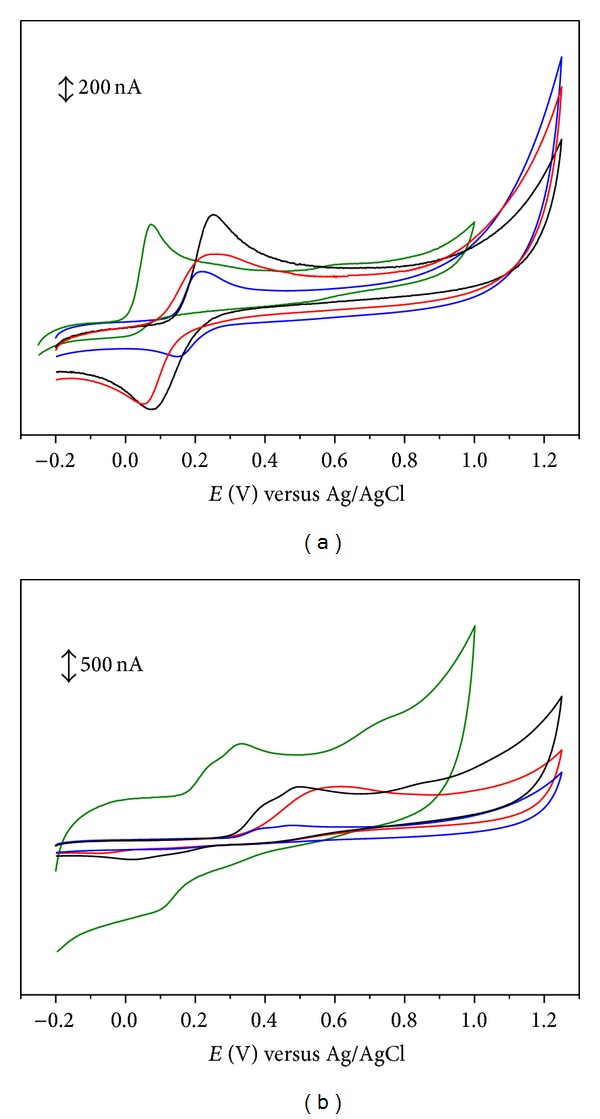
Cyclic voltammograms for 0.1 mM solutions of (a) (black line) caffeic acid, (blue line) 5-bromocaffeic acid, (green line) 3,4,5-trihydroxycinnamic acid, and (red line) hydrocaffeic acid and (b) (black line) ferulic acid, (blue line) 5-bromoferulic acid, (green line) sinapic acid, and (red line) hydroferulic acid, in physiological pH 7.3 supporting electrolyte. Scan rate: 50 mV s^−1^.

The introduction of a second hydroxyl group at the *ortho* position (catechol) leads to a significant decrease of the redox potentials (Tables 1 and 4). DP voltammogram of caffeic acid presents only one well-defined anodic peak at physiological pH (Figure 1) [7–9]. The oxidation peak observed was related to the oxidation of catechol group. Cyclic voltammograms (Figure 2) obtained showed that caffeic acid is reversibly oxidized in solutions of pH up to 5.5 [33]. The electrochemical oxidation follows a mechanism involving only one step with the transfer of two electrons and two protons. The oxidation product was identified as being the corresponding *o*-quinone [6–9, 28, 29, 31, 33]. For pHs higher than 5.5 caffeic acid *o*-quinone becomes unstable, with a chemically homogeneous irreversible reaction occurring [33]. 

Substitution of the 3-hydroxyl group of caffeic acid by a methoxy group (ferulic acid, Table 1) considerably shifts the redox potential toward more positive values (Table 4). The differential pulse voltammetric study of ferulic acid revealed the presence of two convolved anodic peaks at physiological pH (Figure 1). The oxidation peaks were related to the oxidation of the phenolic group present in the structure. The results have been interpreted by assuming that ferulic acid oxidation takes place by electron transfer for both free and adsorbed forms [7–9]. The free form corresponds to the first peak while the strongly adsorbed form, which is consequently stabilized, is oxidized at a more anodic potential. Cyclic voltammograms obtained for ferulic acid have also shown two convolved anodic peaks (Figure 2) [6–9, 28, 29, 34]. The proposed mechanism is similar to that described previously to *p*-coumaric acid.

It has been generally accepted that the introduction of substituents in the aromatic ring of hydroxycinnamic acids could have a positive influence on the formation and lifetime, through a stabilizing effect, of the corresponding phenoxyl radical with a subsequent enhancement of their antioxidant activity. Therefore, it would be expected that the introduction of a third hydroxyl (3,4,5-trihydroxycinnamic acid), a methoxyl (sinapic acid), or a bromide (5-bromocaffeic and 5-bromoferulic acids) substituent in position-5 of the aromatic ring of the cinnamic acid could affect the formation and/or stabilization of the radical intermediate causing a decrease of the oxidation potential. The differential pulse voltammetric study of 3,4,5-trihydroxycinnamic acid (Table 1) revealed the existence of two well-defined anodic waves at physiological pH (Figure 1) [11]. For 5-bromocaffeic acid, one well-defined anodic peak was observed at physiological pH (Figure 1) [7]. The oxidation waves of these hydroxycinnamic compounds have been related to the oxidation of catechol group present in their structure [7, 11]. Similar to caffeic acid, it has been assumed that 3,4,5-trihydroxycinnamic and 5-bromocaffeic acids are oxidized to quinone via semiquinone form. The voltammetric behaviour of sinapic and 5-bromoferulic acids (Table 1) has been also studied at a glassy carbon working electrode using differential pulse voltammetry (Figure 1) [7, 10, 29]. Both compounds present two convolved anodic peaks at physiological pH, using differential pulse voltammetry. The oxidation peaks have been related to the oxidation of the phenolic group present in their structures. As for ferulic acid, these results may be interpreted by assuming that the oxidation of sinapic and 5-bromoferulic acids takes place by electron transfer from both free and adsorbed forms. The presence of a methoxyl group in position-5 causes a cathodic shift on the oxidation potential, with respect to ferulic acid (Table 4). 

In summary, it was concluded that a considerable decrease in the peak potential has only been observed for 3,4,5-trihydroxycinnamic acid and sinapic acid relative to caffeic and ferulic acids, respectively (Table 4). The introduction of a bromide group in position-5 did not significantly change the oxidation potentials, when compared to caffeic and ferulic acids. From the data acquired so far, some relevant conclusions can be addressed: the presence of electron withdrawing groups of halogen type in an *ortho* position to a hydroxyl group does not influence noticeably the redox potential of hydroxycinnamic acids. This type of substituent appears not to disturb the stability of the phenoxyl radical formed as well as its stabilization through intramolecular interactions. On the other hand, lower potential values can be attained, resulting from the increased stabilization of the phenoxyl radical, if electron-donating hydroxyl or methoxyl substituents are present in the aromatic ring.

Another structural feature that seems to have a positive effect on the reducing properties of hydroxycinnamic acids is the ethylenic side chain that connects the aromatic ring to the carboxylic acid [4, 5, 35]. In fact, it is assumed that an additional stabilization of the phenoxyl radical can occur due to its conjugation effect. Thus, the study of hydrocaffeic and hydroferulic acids (Table 2) can contribute to shed a light on this subject. The DP voltammograms of hydrocaffeic and hydroferulic acids present only one well-defined anodic peak at physiological pH (Figure 1) [8]. The cyclic voltammogram obtained for hydrocaffeic acid (Figure 2) is characteristic of an electrochemical reversible reaction showing only one anodic peak and one cathodic peak on the reverse scan [8]. The oxidation peak observed has been related to the oxidation of catechol group [8]. The redox potential obtained for hydrocaffeic acid has been found to be lower than that obtained for caffeic acid (Table 4). On the other hand, the CV study of hydroferulic acid (Figure 2) showed the occurrence of one irreversible peak at a potential slightly higher than ferulic acid (Table 4). So, the information obtained for these two hydroxycinnamic acids was not conclusive and the contribution of conjugation remain unclear.

**Figure 3:**
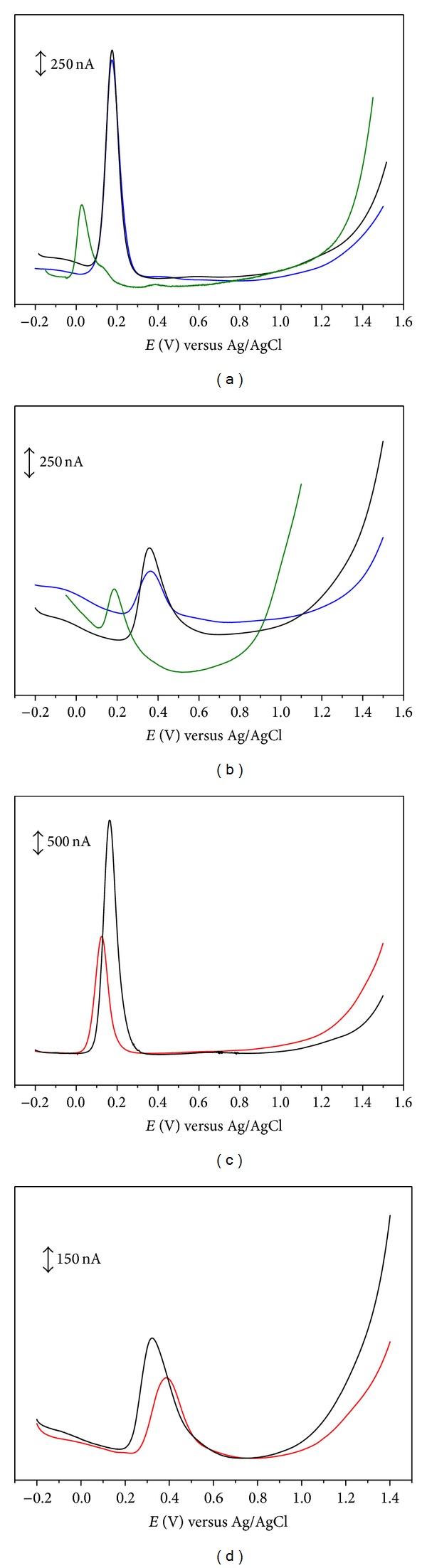
Differential pulse voltammograms for 0.1 mM solutions of (a) (black line) ethyl caffeate, (blue line) ethyl 5-bromocaffeate, (green line) ethyl 3,4,5-trihydroxycinnamate acid; (b) (black line) ethyl ferulate, (blue line) ethyl 5-bromoferulate, (green line) ethyl sinapate; (c) (black line) caffeoylhexylamide, (red line) hydrocaffeoylhexylamide; and (d) (black line) feruloylhexylamide, (red line) hydroferuloylhexylamide, in physiological pH 7.3 supporting electrolyte. Scan rate: 5 mV s^−1^.

Furthermore, several studies have been conducted to evaluate the influence of the modification of carboxylic function by alkyl groups, of different length and nature linked by ester or amide moieties, on the antioxidant efficiency of hydroxycinnamic acids. The voltammetric analysis of a series of caffeic, hydrocaffeic, ferulic, hydroferulic and sinapic acid derivatives has been performed (Tables 1–3, Figure 3) [5, 7–9, 29, 36, 37]. In general, the voltammetric behaviour of these derivatives was similar to that observed for the parent compounds. The introduction of an alkylamide or an alkylester group in the side chain did not significantly change the redox potentials (Table 4). In fact, from the voltammetric data it can be stated that esterification or amidation of the carboxylic acid group of hydroxycinnamic acids by alkyl chains does not significantly change the oxidation potential. Nevertheless, the slight changes observed could be satisfactory to explain the antioxidant activity differences found between HCAs and their esters or amides.

## 5. Relationship between Redox Potential and Antioxidant Properties

Total antioxidant capacity (TAC) assays have been often used to determine the hierarchy of radical-scavenging abilities of potential phenolic antioxidant compounds that work either through electron- or H-donating mechanisms [24]. Thus, to evaluate the radical-scavenging ability of the phenolic acids and their derivatives total antioxidant capacity assays, namely, DPPH^•^ (2, 2′-diphenyl-1-picrylhydrazyl radical), are often used [8, 38]. DPPH^•^ method has the advantage of establishing a ranking hierarchy of antioxidant activity as some factors that cause interference in other model systems, such as metal chelation and/or partitioning abilities, are absent [39, 40]. 

The radical scavenging activity of hydroxycinnamic acids and derivatives on DPPH radical are depicted in Table 4. Trolox, a water-soluble vitamin E analogue, was used as reference standard. In general, all the compounds have been found to be radical scavengers, in a dose-dependent manner, and present better radical-scavenging activity than the reference antioxidant (trolox). 

The antioxidant activity of cinnamic acids and derivatives is dependent on their molecular structure. The main structural features responsible for the activity are the presence of a phenolic group and the capacity of the compound to stabilize the resulting phenoxyl radicals.

Taken together, the results obtained reveal the existence of a relation between redox potentials of the hydroxycinnamic acids and derivatives and antioxidant activity (DPPH assay) (Table 4). The correlation between the redox potential of antioxidants, especially phenolics, and its antioxidant activity is already documented in the literature, but still the subject of intense research [8, 25, 26]. The radical scavenging activity data acquired for hydroxycinnamic acids and derivatives is in good agreement with the expected activities of this type of phenolic systems: it is higher when the redox potential is lower (as seen in the caffeic series, Table 4). The presence of a catechol group leads to an increase of the activity due mainly to resonance stabilization of the phenoxyl radical intermediate with subsequent *ortho*-quinone formation. The absence of a double bond in the side chain (as seen in hydrocaffeic series, Table 2) gives rise to compounds with a lower redox potential and an increase in the antioxidant activity. However, the methoxylation of the *meta*-phenolic group (ferulic and hydroferulic series, Tables 1 and 2) leads to an increase of redox potential and a decrease of the antioxidant activity. In this type of systems, the ethylenic double bond displays a negative effect in both properties. It is important to notice that the introduction of another methoxyl group in an *ortho* position to a hydroxyl group (sinapic acid series, Table 1) results in an increase of the antioxidant activity and a decrease of the redox potential relative to ferulic acid. 

In addition, it was shown that the introduction of a halogen substituent in an *ortho position* to a phenolic group of the hydroxycinnamic acids (or their linear monoalkyl esters) has no influence either in the redox potential or the antioxidant activity (Tables 1 and 4). The presence of this electron withdrawing group (*σ*-acceptor and a weak *π*-donor) in an *ortho position* to a hydroxyl group appears not to disturb the stability of the phenoxyl radical formed as well as its stabilization through intramolecular interactions. 

The insertion of an alkyl side chain either by using ester or amide as linkers has been found to have different effects on the antioxidant activity on phenolic cinnamic acid systems. The data obtained so far reveal that caffeic acid derivatives have higher DPPH radical scavenging activities and lower redox potentials than caffeic acid itself, a result that seems to be dependent on the extension, or type, of the ester or amide side chain (Tables 1 and 4). Opposite tendency was obtained for the ferulic and sinapic alkyl esters. In fact, the alkyl derivatives have lower antioxidant activities, a fact that is in accordance with electrochemical data (Tables 1 and 4). 

From the overall results, one can assume that the effect of the alkyl ester side chain in hydroxycinnamic systems is strongly related to the number of hydroxyl groups and the aromatic substitution pattern. In fact, it seems to be intimately connected with the different oxidation mechanisms and capacity of stabilization of intermediates. Caffeic acid and its derivatives operate *via* quinone intermediate and ferulic and sinapic derivatives *via* semiquinone intermediates. The latter are further stabilized by the inductive and mesomeric effects of the methoxyl functions. These overall effects can surpass the mesomeric/inductive influence of the ethylenic side chain.

As concluding remarks, we can endorse that a general antioxidant relationship statement cannot be performed for hydroxycinnamic acids and derivatives: each series of compounds is a case and the structural modification must be cautiously analyzed as the activity is a consequence of the sum of steric, mesomeric, and inductive effects on the system. 

In summary, one can say that redox potentials were found to rule the antioxidant activities of this type of compounds, being an effective approach for the rational design of new antioxidant agents as well as for the understanding of their mechanism of action.

## 6. Electrochemistry in a Real World

Electrochemical methods are a very versatile tool that can be applied not only in qualitative but also quantitative analysis. Hydroxycinnamic acids and derivatives need to be determined in real samples at low (*μ*g/L) or high concentration levels [41]. Electrochemical methods, using voltammetric or amperometric detection with different cell designs, can offer excellent sensitivity and selectivity by varying the working potential. Although several methods have been developed to monitor the levels of phenolic compounds in wine [42–45], none of them is able to specify the reducing capacity of the (poly)phenols present or to distinguish between catechol and galloyl-containing (poly)phenols, which are easy to oxidize (e.g., caffeic acid) and those that are more difficult to oxidize (e.g., coumaric acid). Taking into account that (poly)phenols are electrochemically active compounds and can be oxidized at inert electrodes, electrochemical methods can be used to achieve this goal. In fact, cyclic voltammetry has been extensively applied to characterize a variety of wine phenolics [28, 46–54]. This technique has been used to discriminate and to estimate the total polyphenolic content in wine [47–49] and to study the antioxidant activity providing a measure of the “Redox spectra of Wines” [50, 51]. Moreover, this type of methods has been used to evaluate the “oxidation status” of white wines [52] and perceived astringency of red wines [53].

Electrochemical methods appear as simple and sensitive methods giving good estimations of the global content of polyphenols [54, 55]. As hydroxycinnamic acids and derivatives are of natural origin and are present in a diversity of matrices, namely grapes and wine (red or white), beer, green coffee beans and coffee, spices, vegetables, and blueberries, one can envision a flourishing application of electrochemical methods [56–61]. These techniques will be capable of giving the global amount of (poly)phenols and of characterizing new compounds. In the near future, these methods will be applied transversally in different areas, such as food and pharmaceutical industries, namely for the discovery and/or detection of new antioxidants. 

## Figures and Tables

**Table 1:** Hydroxycinnamic acids and ester derivatives.

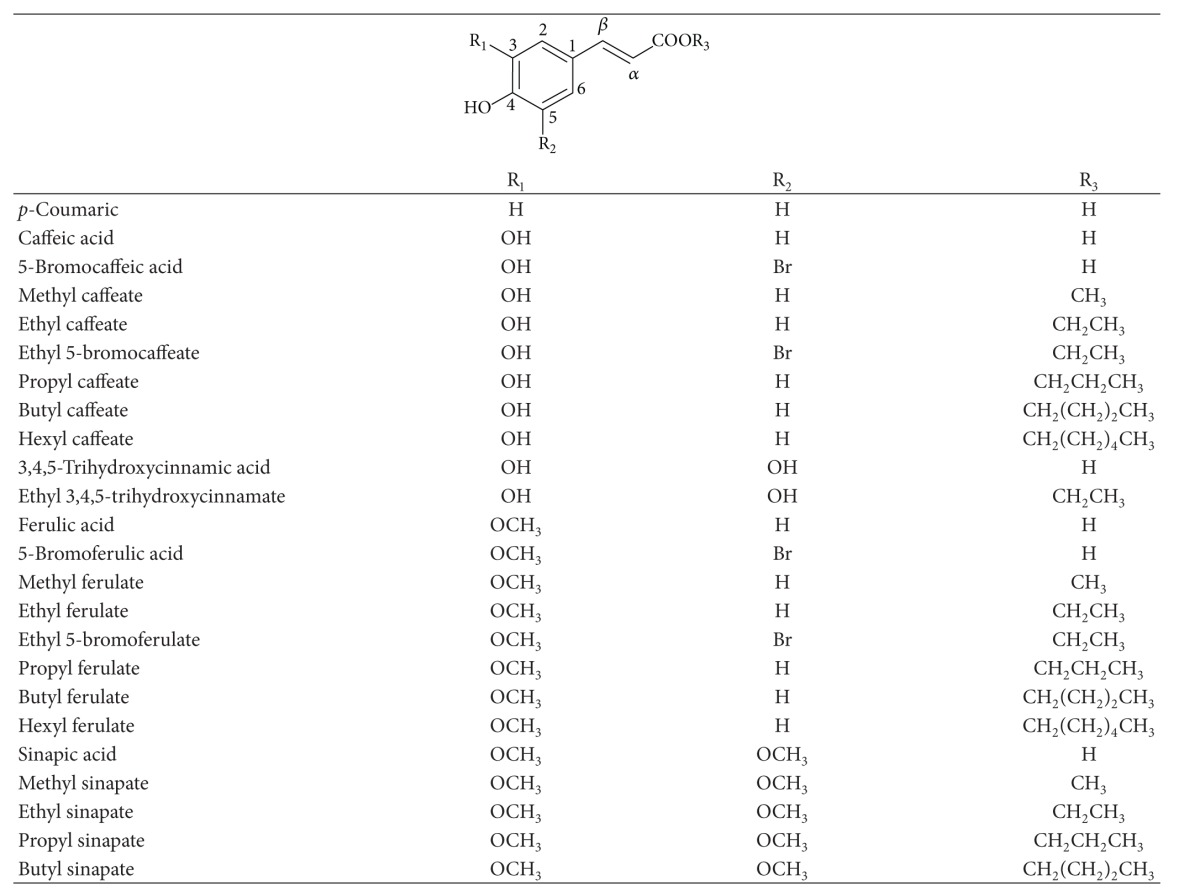

**Table 2:** Hydroxyhydrocinnamic acids and ester derivatives.

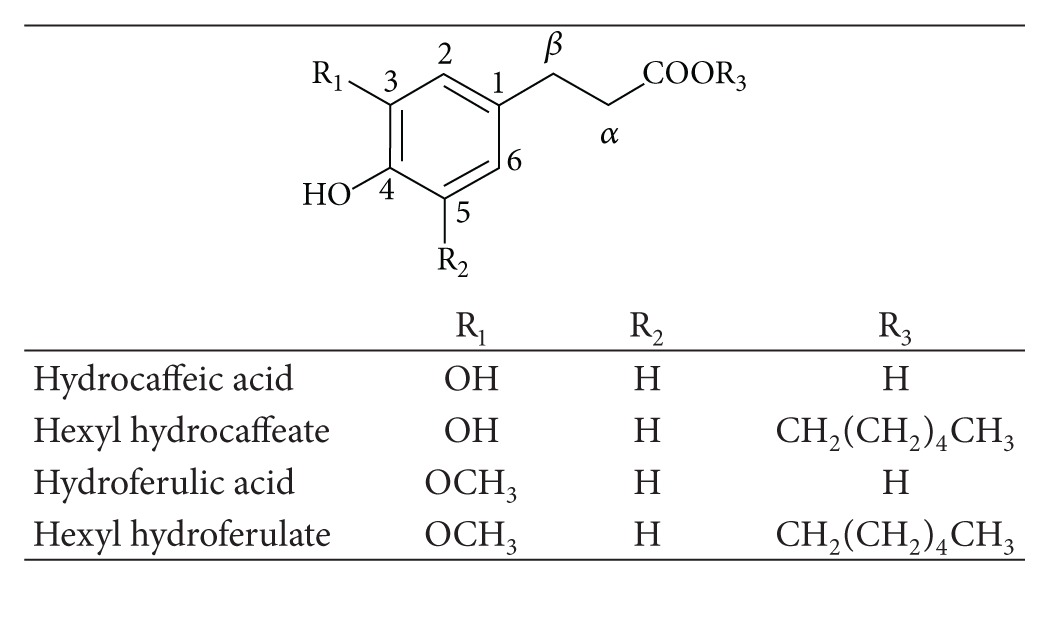

**Table 3:** Hydroxy(hydro)cinnamic amide derivatives.

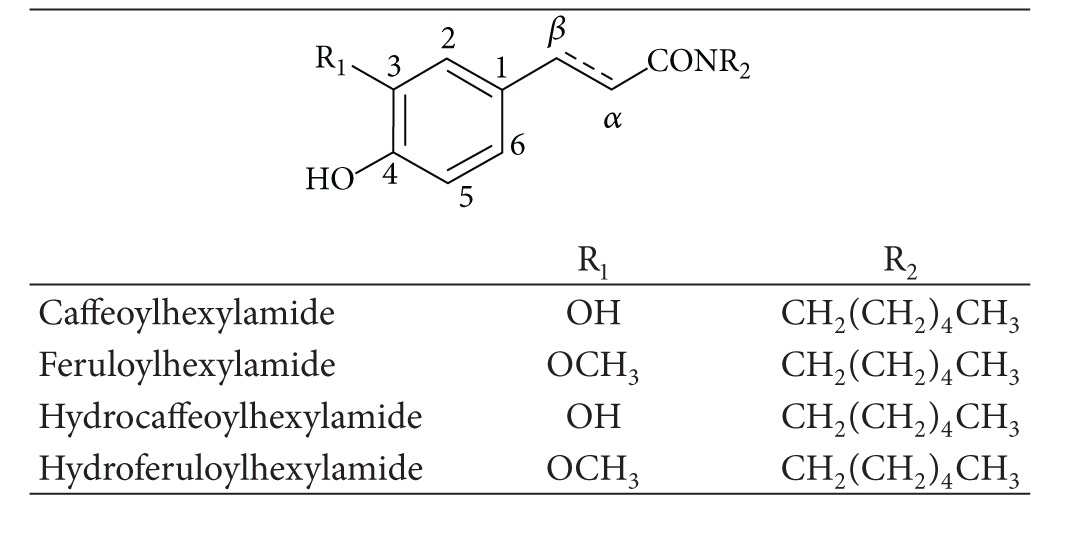

**Table 4:** Redox potentials and antioxidant activity of hydroxycinnamic acids, ester and amide derivatives.

Compound	MW (gmol^−1^)	DPPH (TE)*	DPPH (IC_50_)^∗†^	*E*_*p*_ (mV)	Reference
*p*-Coumaric	164.16		>100	+736	[6]
Caffeic acid	180.16	1.29	16.6	+183	[7, 8]
Hydrocaffeic acid	182.18	2.05	15.4^#^	+139	[8]
5-Bromocaffeic acid	259.05	1.01		+182	[7]
Methyl caffeate	194.18		14.0	+165	[9]
Ethyl caffeate	208.21		13.5	+170	[9]
Ethyl 5-bromocaffeate	287.11	0.938		+174	[7]
Propyl caffeate	222.24		14.5	+173	[9]
Butyl caffeate	236.26		14.1	+176	[9]
Hexylcaffeate	264.32	0.970		+175	[8]
Hexylhydrocaffeate	266.34	0.990		+125	[8]
Caffeoylhexylamide	263.34	1.11		+162	[8]
Hydrocaffeoylhexylamide	265.35	1.00		+125	[8]
Ferulic acid	194.19	0.781	44.6	+335; +447	[7]
Hydroferulic acid	196.20		84.0^#^	+410	[8]
5-Bromoferulic acid	273.08	0.808		+335; +442	[7]
Methyl ferulate	208.21		74.7	+375	[9]
Ethyl ferulate	222.24		66.7	+370	[9]
Ethyl 5-bromoferulate	301.13	0.558		+365	[7]
Propyl ferulate	236.26		64.1	+364	[9]
Butyl ferulate	250.29		56.3	+343	[9]
Hexylferulate	278.35			+328	[8]
Hexylhydroferulate	280.36			+434	[8]
Feruloylhexylamide	277.36			+322	[8]
Hydroferuloylhexylamide	279.38			+388	[8]
Sinapic acid	224.21	0.862	32.2	+188; +295	[10]
Methyl sinapate	238.24		48.7	+219	[10]
Ethyl sinapate	252.26		51.9	+189	[10]
Propyl sinapate	266.29		50.6	+182	[10]
Butyl sinapate	280.32		50.1	+190	[10]
3,4,5-Trihydroxycinnamic acid	196.16		11.8	+11; +384	[11]
Ethyl 3,4,5-trihydroxycinnamate	224.21		15.1	+26; +133	[11]

*The results of DPPH assays are usually expressed as TEAC (trolox equivalent antioxidant capacity) or IC_50_ (concentration which is required to scavenge 50% of DPPH free radicals). These values are not convertible.

^†^IC_50_ is presented in *μ*mol L^−1^.

^#^Unpublished results.
